# A Novel Continuously Recording Approach for Unraveling Ontogenetic Development of Sleep-Wake Cycle in Rats

**DOI:** 10.3389/fneur.2019.00873

**Published:** 2019-08-13

**Authors:** Guang-Fu Cui, Min Hou, Yu-Feng Shao, Hai-Lin Chen, Jin-Xian Gao, Jun-Fan Xie, Yu-Nong Chen, Chao-Yu Cong, Feng-Qiu Dai, Yi-Ping Hou

**Affiliations:** ^1^Departments of Neuroscience, Anatomy, Histology, and Embryology, Key Laboratory of Preclinical Study for New Drugs of Gansu Province, School of Basic Medical Sciences, Lanzhou University, Lanzhou, China; ^2^Department of Anatomy, Gansu University of Traditional Chinese Medicine, Lanzhou, China

**Keywords:** polysomnographic recording, sleep-wake states, ontogeny of sleep, infant rats, milk-feeding, temperature-controlled

## Abstract

Sleep-wake development in postnatal rodent life could reflect the brain maturational stages. As the altricial rodents, rats are born in a very undeveloped state. Continuous sleep recording is necessary to study the sleep-wake cycle profiles. However, it is difficult to realize in infant rats since they rely on periodic feeding before weaning and constant warming and appropriate EEG electrodes. We developed a new approach including two types of EEG electrodes and milk-feeding system and temperature-controlled incubator to make continuously polysomnographic (PSG) recording possible. The results showed that there was no evident difference in weight gaining and behaviors between pups fed through the milk-feeding system and warmed with temperature-controlled incubator and those kept with their dam. Evolutional profiles of EEG and electromyogram (EMG) activities across sleep-wake states were achieved perfectly during dark and light period from postnatal day (P) 11 to P75 rats. The ontogenetic features of sleep-wake states displayed that the proportion of rapid eye movement (REM) was 57.0 ± 2.4% and 59.7 ± 1.7% and non-REM (NREM) sleep was 5.2 ± 0.8% and 4.9 ± 0.5% respectively, in dark and light phase at P11, and then REM sleep progressively decreased and NREM sleep increased with age. At P75, REM sleep in dark and light phase respectively, reduced to 6.3 ± 0.6% and 6.9 ± 0.5%, while NREM correspondingly increased to 37.5 ± 2.1% and 58.4 ± 1.7%. Wakefulness from P11 to P75 in dark phase increased from 37.8 ± 2.2% to 56.2 ± 2.6%, but the change in light phase was not obvious. P20 pups began to sleep more in light phase than in dark phase. The episode number of vigilance states progressively decreased with age, while the mean duration of that significantly increased. EEG power spectra in 0.5–4 Hz increased with age accompanied with prolonged duration of cortical slow wave activity. Results also indicated that the dramatic changes of sleep-wake cycle mainly occurred in the first month after birth. The novel approaches used in our study are reliable and valid for continuous PSG recording for infant rats and unravel the ontogenetic features of sleep-wake cycle.

## Introduction

The ontogenetic hypothesis of sleep, proposed 50 years ago, postulates that early developmental sleep is essential for maturation of fundamental brain function (1). Daily sleep amounts are highest early in development across multiple species (2–5) that lead to promote normal brain development that give rise to adult critical behaviors for learning, memory consolidation, emotional processing and species propagation (4, 6, 7). Human studies have showed that impaired sleep during early periods of development can have severe and longlasting consequences such as cognitive, attentional, and psychosocial problems (8–10).

In all species studied so far, measures of sleep changes throughout development are fundamental ways for unraveling which regions of brain are most susceptible to sleep perturbations early in life. Sleep electroencephalogram (EEG) in humans and cortical EEG recordings in animals provide unique *in vivo* opportunities to observe regional changes in brain activity over the course of cortical maturation. In the human being, the distinct electrical patterns associated with the different sleep states begin to emerge approximately at 28 weeks' gestation age. By 30 weeks' gestation, the EEG patterns of rapid eye movement (REM) and non-REM (NREM) sleep (also named as active sleep (AS) and quiet sleep (QS) in the infant, respectively) appear but are not continuous (11). The ontogenetic changes of sleep in animal life are similar to those in humans (4, 12). The rat is an altricial born in a far less mature condition than humans and its cortical maturity during the first postnatal week corresponds to that of the young premature human brain (13–15). Thus, rats are good models to study the development of the sleep-wake cycle and its EEG rhythms because more immature stages of these processes can be studied in postnatal life when they are more experimentally accessible (16).

In recent studies, the sleep-wake states in rat pups are identified by combining visual observation with measurements of muscle activity and EEG recordings (2, 17–20). However, most of these studies observed and recorded intermittently, which means they cannot get 24 h continuous recordings, and cannot elaborated the dramatic changes and circadian development during the whole day in early life. The limitations of the early methods for intermittent recordings are largely due to periodic feeding before weaning and keeping warm during long-term sleep recording in pups after maternal separation. The sensitivity of pups to limosis and improper temperature around recording environment easily produces significant changes in sleep-wake pattern (21, 22). Furthermore, an inadequacy of the conventional screws as EEG electrodes for polysomnographic (PSG) recording are implanted into the neonatal rat skull and wired with a relatively large head plug cemented onto the small skull surface that is soft, fragile, and rapid growing. Therefore, it is necessary to make light and suitable EEG electrodes which could effectively achieve EEG signals from young pups.

We have recently developed the novel approaches including a milk-feeding system for the pre-weaned pups, a temperature-controlled incubator, and two types of EEG electrodes and well-matched plug according to the pup age for continuously (24 h/day) PSG recording that overcomes the above limitations of conventional methods. Moreover, we carried out a periodic stimulation on the anogenital region and grooming for the pre-weaned pups in order to induce micturition and defecation (a process usually performed by the lactating dam), and to minimize the stress response due to maternal deprivation (2, 23, 24). The present study describes the new PSG recording methods in detail and offers ontogenetic features of sleep-wake cycle in rats from postnatal day (P) 11 to P75.

## Materials and Methods

### Animal Preparation

Adult male and female Sprague-Dawley rats (6–8 weeks old, weighing = 250 ± 35 g) were purchased from the Experimental Animal Center of Lanzhou University (Lanzhou, PR China). A male with two female rats were housed in a plastic cage (485 mm L × 350 mm W × 225 mm H) for mating and kept in an automatically controlled room in a 12:12-h light/dark cycle (lights on 8:00–20:00 h, illumination intensity = 100 lx) at an ambient temperature (23 ± 1°C) and 50% relative humidity with food and water available *ad libitum*. The mating procedure was repeated on successive days until copulation and confirmed on the basis of vaginal plug formation (monitored every morning). The pregnant rats were individually housed in cages and checked twice daily until birth. The day of birth was defined as P0, and 10 pups of a litter were generally kept in their dam and their behaviors were monitored by an infrared video camera. A total of 19 pregnant rats were used in this study, and 89 of offspring were successfully used to record sleep-wake states. All animals were cared for, and experiments were conducted in accordance with the National Institutes of Health Guide for the Care and Use of Laboratory Animals (2011 revision). The experimental protocol was approved by the Ethics Committee of Lanzhou University (permit number: SCXK Gan 2018–0002, Lanzhou, PR China). All possible efforts were made to reduce the number of animals used and discomfort to the animals.

### EEG and EMG Electrodes and Their Implantation

**Figure 1:**
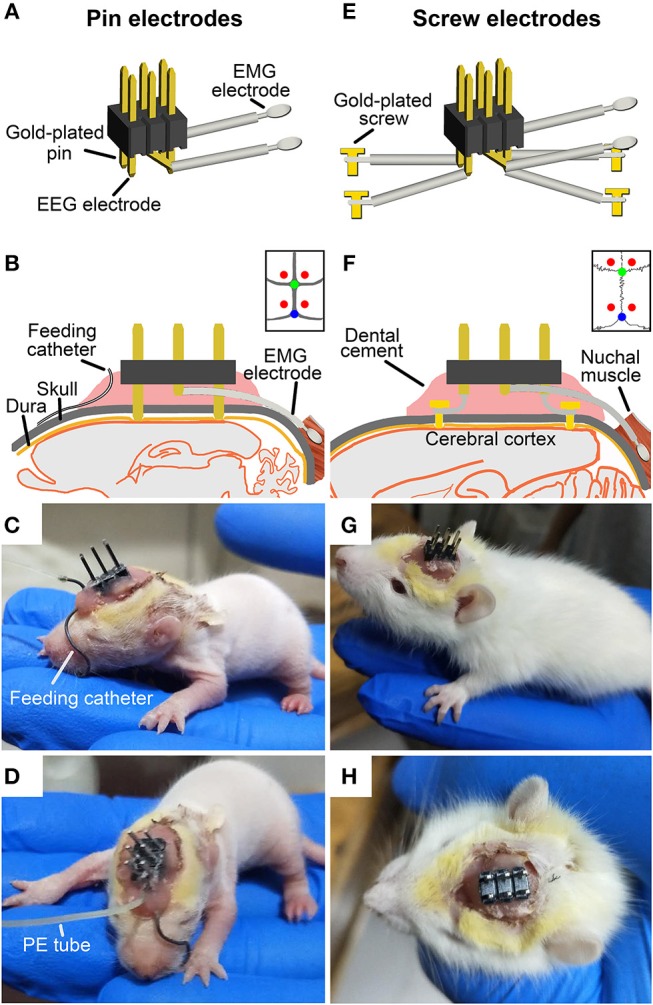
Schematic showing the implantation of two types of EEG and EMG electrodes with or without a milk-feeding catheter respectively, in ≤P16 **(A–D)** and ≥P17 pups **(E–H)**. For ≤P16 pups, 4 gold-plated pins **(A)** as EEG electrodes were directly implanted through skull to contract with the dura (**B**, implanted sites are shown by red points in insert), and 2 silver loops filled tin as EMG electrodes were inserted into nuchal muscles. A metal milk-feeding catheter **(C)** connected with PE tube **(D)** was amenably placed along left angulus oris into side of oral cavity. For ≥P17 pups, 4 gold-plated screws as EEG electrodes **(E)** were screwed through skull into the dura **(F**, screwed sites are shown by red points in insert) and a pair of silver loops as EMG electrodes was inserted into nuchal muscles. **(G,H)** Respectively, show side and top view of the plug fixed chronically to skull with dental cement. Green and blue points in insert of **(B,F)** respectively, show bregma and lambda.

In order to effectively acquire EEG and electromyogram (EMG) signals and to reduce weight of electrodes borne by pups, two types of EEG electrodes were used respectively, for ≤P16 and ≥P17 pups. For ≤P16 pups, 4 gold-plated pins (top of pin = 0.5 × 0.5 mm) served as EEG electrodes were assembled within a 6-pin pedestal (weighing = 0.3 g). As shown in Figure 1A, the interval between left and right pins and between anterior and posterior pins respectively, is 2.54 and 5.08 mm. For ≥P17 pups, 4 gold-plated screws (diameter = 1 mm) served as EEG electrodes were connected with insulted silver-plated copper wires and soldered into a 6-pin pedestal socket (Figure 1E). One end of a pair of 25 mm silver wires (diameter = 0.5 mm) insulated with fluorinated ethylene propylene was exposed to 5 mm and then looped and filled with soldering tin served as EMG electrodes, and the other end was soldered into the pedestal socket (Figure 1A). Finally, the exposed welding points were covered and insulated by hot melt adhesive for preventing possible surgery-induced conduct electricity from neighboring wires. The electrical continuity between the electrodes and the outputs of pedestal socket was checked with a digital multimeter before the implantation of EEG and EMG electrodes.

Under isoflurane anesthesia (1%, flow rate of 0.4 L/min; R510-22, RWD Life science Co. Ltd, Shenzhen, PR China), pups were prepared for aseptic surgery, and secured in a SR-6R stereotaxic frame (Narishige, Tokyo, Japan) on a homeothermic heating mat (37°C, ThermoStar, 69020, Life science Co. Ltd., Shenzhen, PR China). Four pins EEG electrodes were directly inserted onto the dura mater through the two pairs of skull holes that are corresponded to the interval between the 4 pins, and were located, respectively, in the frontal (1.27 mm lateral and 1 mm anterior to the bregma) and parietal (1.27 mm lateral to the midline and 4.08 mm posterior to the bregma) cortices (Figure 1B). Four gold-plated screws EEG electrodes were screwed through the similar coordinates on the skull onto the dura mater (Figure 1F). The EMG electrodes were bilaterally placed into the nuchal muscles. The pedestal socket was chronically fixed to skull with dental cement (Figures 1C,G).

### Feeding Catheter and Its Installation

Based on our explored experiments and previous report (25), the ≤P16 pups need to be lactated for getting adequate nutrition. A feeding catheter was made of a stainless-steel tube (internal diameter = 0.5 mm, length = 31 mm), one end of which was obtuse for inserting oral cavity, and the other end of which was connected with a pliable PE tube for pumping milk.

Under anesthesia, the oral end of feeding catheter was simultaneously inserted along left angulus oris into ipsilateral oral cavity near buccal mucosa for 2 mm after EEG and EMG electrodes implantation. The rest of feeding catheter was curved along the curvature of skull, and the end connected with PE tube was reached and fixed with dental cement to the anterior of pedestal socket (Figures 1C,D).

### Care for Young Pups After Surgery

**Figure 2:**
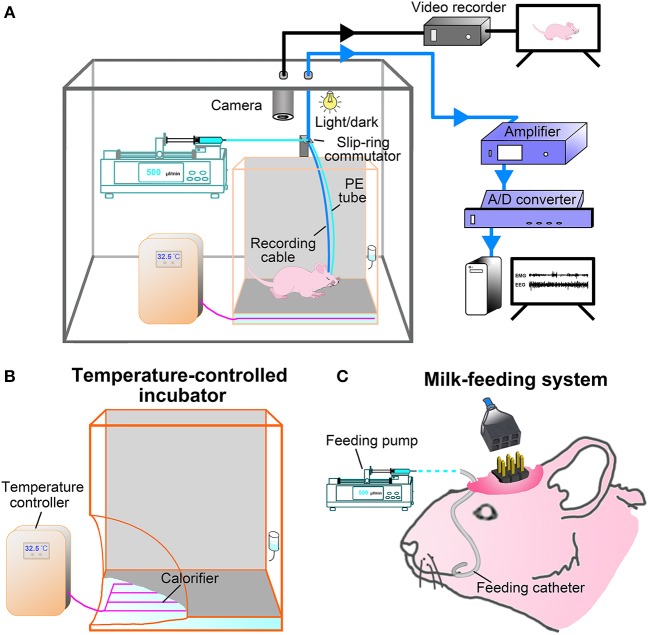
Experimental setup. **(A)** Constructing illustration shows sleep-wake states and behavior recording and analyzing setup for freely moving infant rats. Pups were kept in a temperature-controlled incubator in which the ambient temperature was set according to its age and controlled by temperature sensor that automatically switched on and off the calorifier **(B)**, and their behaviors on a 12:12-hlight/dark cycle in the sleep recording chamber was monitored with an infrared camera and stored with video recorder (black line). The ≤P16 pups were fed once per hour by a milk-feeding system which was consisted of a programmable infusion pump and a conterminous PE tube connected to feeding catheter **(C)**. The ≥P17 pups freely accessed to rat chow and milk or water. EEG and EMG signals were transmitted through a flexible cable connected with slip ring to an amplifier, digitized with an A/D convertor, and stored and analyzed with Spike 2 scripts (CED, UK) installed in PC (blue line).

After surgery, the pups were placed singly in a temperature-controlled incubator and fed by milk-feeding system within a sound-attenuated, ventilated and electrically isolated sleep-recording chamber, and allowed to recover for 36 h (Figures 2A,B). The incubator (300 mm L × 300 mm W × 400 mm H) is made of acrylics, and its base part was immersed in temperature-controlled water bath. The pups were placed on a diaper covered the bottom of the incubator during the recovery and sleep-wake recording. The thermal environment was controlled with a temperature sensor that automatically switched on and off the calorifier (Figures 2A,C), and it was set by the age of P9-P20 (Table 1) based on our explorative studies and previous reports (24, 26, 27).

**Table 1:** The parameters of formula milk infusion and ambient temperature set at each age.

**Postnatal days**	**Formula milk infusion**		**Ambient temperature (**°**C)**
	**Rate (mL/min)**	**Volume (mL/day)**	
9	0.250	6.0	36
10	0.300	7.2	36
11	0.350	8.4	35
12	0.375	9.0	34
13	0.400	9.6	33
14	0.500	12.0	32
15	0.500	12.0	31
16	0.625	15.0	30
17	–	–	29
18	–	–	27
19	–	–	25
20	–	–	23

The milk-feeding system was consisted of a computer-assisted infusion pump (KDS210, KD Scientific, MA, USA) and a conterminous PE tube connected to feeding catheter. The feeding pump was programmed to infuse formula milk (20%, Nestle, Lactogen-1, Harbin, PR China) for 1 min per hour, and its delivering rate was set to be almost suckled by pups at each corresponding age (Table 1). The total volume of milk infused at each age was calculated to result in a weight gain similar to that occurs in pups lactated by mater over the same period of time (24) (Table 1). In addition to milk, rat chow soaked with milk was added to pups at P16. Based on our probe trials and previous studies, pups could be weaned at P16 without any major effects on their development (25). When pups were older than P17, standard rat chow, formula milk in bottle for P17-P20 and water for P21-P75 were available *ad libitum*. The pre-weaned pups were also ministered to stimulate anogenital region and groom body with moistened cotton swabs twice a day, so as to induce micturition and defecation and reduce the stress of maternal deprivation. All pups from the same litters with or without the experimental process were weighed everyday, and their movement, hair growth, and eye-opening time were observed to evaluate the adequate nutritional status.

### Sleep-Wake States Recording and Analysis

During recovery and sleep-wake recording, pups were kept in an automatically controlled sleep-recording chamber on a 12:12-h light/dark cycle (lights on 8:00–20:00 h, illumination intensity = 100 lx), and their wake- and sleep-related behaviors were simultaneously monitored and recorded by an infrared video camera above incubator (Figure 2A). After recovery, a 24-h sleep-wake cycle was recorded following pups acclimation to the recording cable connected to a slip-ring commutator for 12 h. In addition, the P21-P75 rats were kept in a barrel (300 mm L × 300 mm W × 400 mm H) within the sleep-recording chamber with an ambient temperature (23 ± 1°C) for recovery after surgery and sleep recording.

EEG and EMG signals were amplified (2000×) and filtered (0.5–30 Hz for EEG and 30-300 Hz for EMG; Model 3500, A-M Systems, WA, USA) and digitized with a resolution of 256 and 128 Hz, respectively, using CED 1401 MK II (Cambridge Electronic Design Limited, Cambridge, UK) (Figure 2A).

The sleep-waking states were defined by the EEG and EMG signal recordings and behaviors monitored with video camera. Using a Spike 2 (CED, Cambridge, UK) script and with the assistance of spectral analysis by the fast Fourier transform (FFT), PSG records were visually scored by 10-s epochs for wake, NREM and REM sleep according to previously described criteria validated for infant and adult rats (2, 20, 28–32). Briefly, wake was identified by the presence of low-voltage fast-EEG and sustaining high-EMG activities coupled with limbs crawling, moving or standing posture with eyes open. NREM sleep marked by continuous high-voltage slow-EEG and low-EMG activities coupled with limbs curling or uncurling immobility with eyes closed. REM sleep characterized by an appearance of theta waves, in addition to low-voltage fast EEG activity, and an occurrence of irregular burst in persistence of low-EMG activity associated with body twitches (phasic and rapid movements of limbs and tail) during pup's immobility with eyes closed.

The changes of cortical EEG power spectra across the sleep-wake states in each postnatal day were computed for consecutive 10-s epochs in the frequency range 0.5 to 30 Hz by FFT using Spike 2 software with a frequency resolution of 0.5 Hz. A window weighting function (Hanning) was applied before FFT performance (33). In addition, the delta (0.5–4 Hz) power in NREM spectrum was analyzed to display the developmental biomarker in EEG power densities. The percentage of delta power densities relative to NREM spectrum that was performed with FFT in all 10-s epochs of NREM without obvious artifact was compared across the considered key postnatal days (P11, P15, P20, P30, and P75).

### Statistics

All data are expressed as means ± SEM. IBM SPSS Statistics for Windows (version 21.0, NY, USA) was employed for the data analysis. The Student's *t*-test was used to compare weight gain at each age between pups fed by formula milk and by their mater. One-way analysis of variance (ANOVA) followed by *post hoc* Fisher's least significant difference (LSD) was used to analyze the developmental changes in delta power in NREM spectrum, and in the amount, episode number and mean duration of vigilance states and state transitions. Statistical significance was considered as *P* < 0.05.

## Results

### Effectiveness of Temperature-Controlled Incubator and Milk-Feeding System on Growth of Young Pups

**Figure 3:**
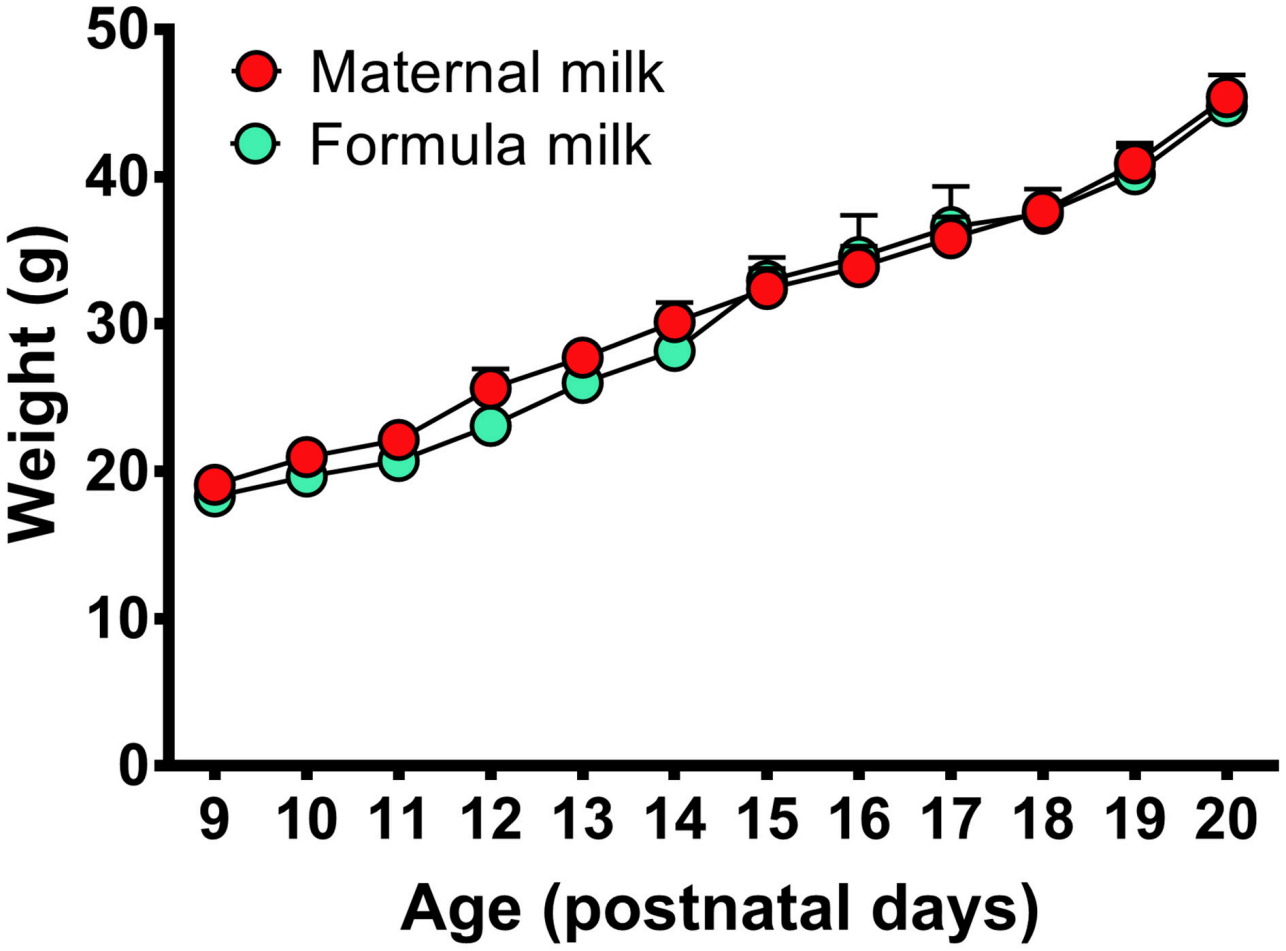
Weight gains in the P9-P20 pups fed respectively, by formula milk and maternal milk. The weight of pups fed with formula milk were not significantly different from that fed with their dam, though the weight gains in the pups fed with formula milk had a slight reduction at P10-P14. Values are means ± SEM (*n* = 5–6 in each age). Data are analyzed by Student's *t*-test.

In this study, a milk-feeding system was used to feed formula milk for P9-P16 pups, and a temperature-controlled incubator was used to keep proper temperature of P9-P20 pups during recovery after surgery and PSG recording according to each age schedule (Table 1). In comparision with the pups fed by their dam, the pups fed with formula milk and warmed with temperature-controlled incubator showed no obvious differences in weight gain at each age (Figure 3) as well as hair growth.

### Behavior Aspects

The behaviors in the rats implanted with EEG and EMG electrodes, and with or without a feeding catheter were monitored through video camera during recovery and PSG recording. The postoperative pups moved freely and easily, presumably because the implant apparatus and pedestal socket linking cable to the commutator were light, soft and pliable. The P9-P11 pups remained eyes closed, and presented distinct behavior patterns. Either they apeared to be awake, with crawling and wiggling movements and tonic extension of neck, or motionless with a muscular hypotonia interrupted by frenquent twitches of the entire body. The twitches were much more intense from P9 to P12. Pup's eyes generally opened during P12-P15, and body twitches were gradually less. The immobile posture with curling or uncurling limbs and without muscular twitches, as presumed NREM sleep, was increased with age. Behavior aspects of sleep and wakefulness at P20 became more adult-like.

### Characteristics of EEG and EMG Activities Across Sleep-Wake States

**Figure 4:**
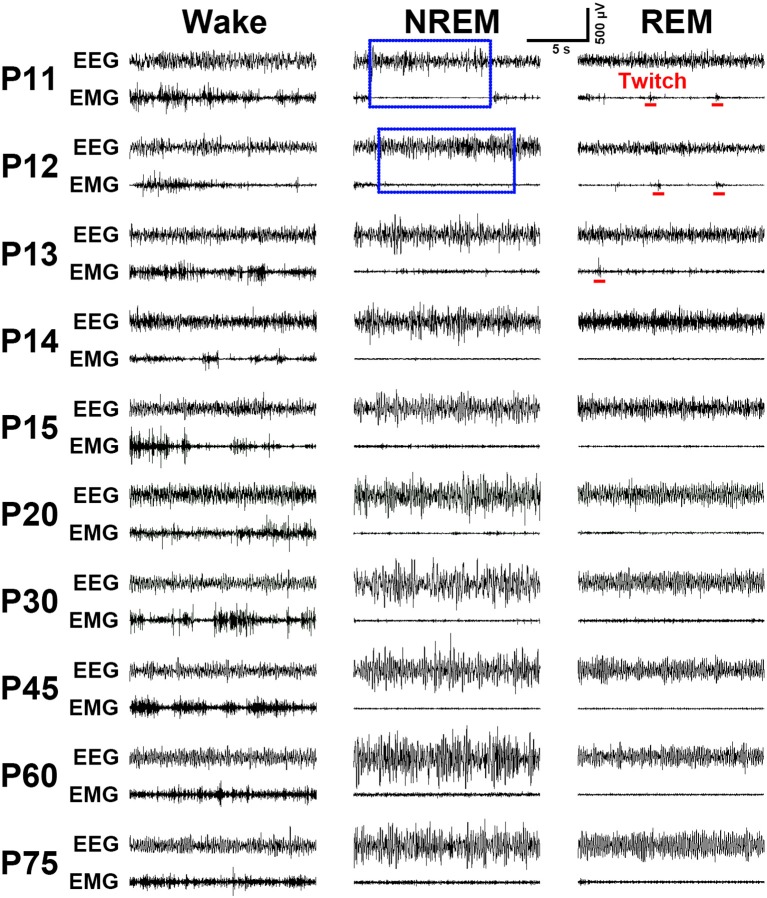
Evolution of EEG and EMG activities in wake, NREM and REM sleep from P11 to P75 rats. Note the EMG bursts during REM sleep denoting the presence of muscular twitches (red bar) in P11-P13 pups. Calibrations: 500 μV, 5 s.

Implantation of the gold-plated pins or screws as EEG electrodes into frontal and parietal dura respectively, for ≤P16 (*n* = 31) and ≥P17 (*n* = 58) rats, and of the silver loops as EMG electrodes into nuchal muscle successfully captured cortical EEG and muscular EMG signals. As summarized in Figure 4, the representative EEG and EMG patterns of each state were clearly distinguished in P11-P75 rats. Overall, P11-P13 pups spent their most time in REM sleep characterized by an appearance of theta waves, in addition to low-voltage fast EEG activity, and an occurrence of nuchal EMG bursts accompanying muscle twitches appearing on a background of very low EMG activity. P14-P20 pups progressively reduced the total time of REM sleep, meanwhile, the occurrence of EMG bursts associated with muscle twitches gradually declined and eventually disappeared. NREM sleep characterized by high-voltage slow-EEG and low-EMG activities in P11-P12 pups were very short and rare. However, its episode number and duration gradually increased during P13-P15. The EEG activity during NREM at P30 was similar to adult (P75). Wakefulness during P11-P75 was characterized by low-voltage fast-EEG activity and sustaining high-EMG activity, though EEG and EMG activities between P11 and P13 showed fragmentation and variation.

### Development of Sleep-Wake Cycle

**Figure 5:**
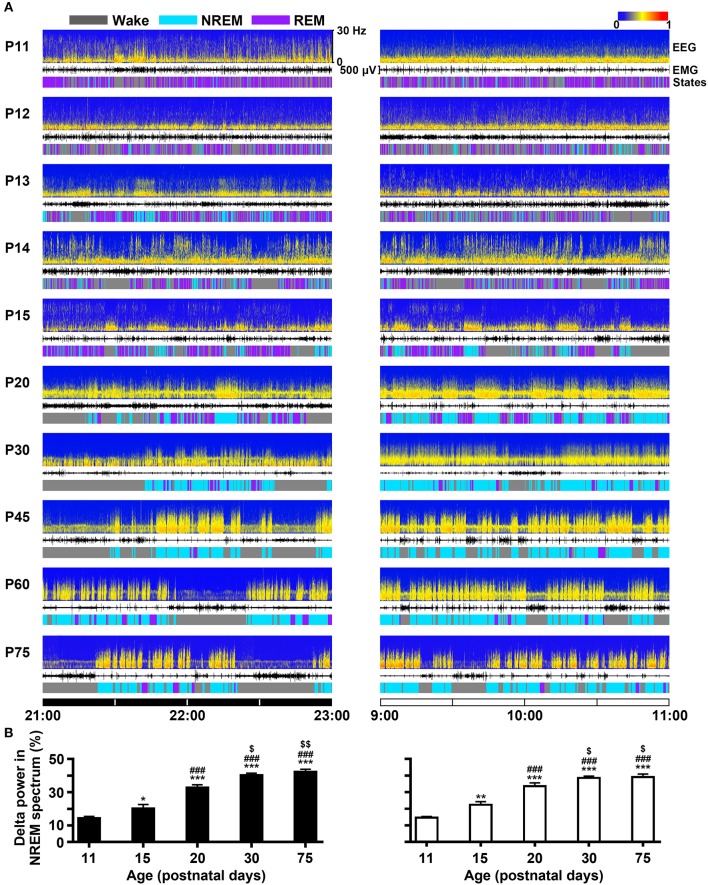
Evolutional profiles of sleep-wake states in dark and light phase from infant to adult rats. **(A)** Representative EEG power spectra (frequency 0–30 Hz), EMG activity (500 μV) and sleep-wake states show the changes during 21:00–23:00 h of dark phase and 9:00–11:00 h of light phase in rats from P11 to P75. **(B)** The analysis of EEG power spectra displayed that the percentage of delta (0.5-4 Hz) power in NREM spectrum at the key postnatal days (P11, P15, P20, P30, and P75) was significantly increased in both dark (21:00–23:00 h) and light (9:00–11:00 h) phases from P11 to P30. Values are means ± SEM (*n* = 5-9 in each age), *P < 0.05, **P < 0.01, ***P < 0.001 compared with P11; ^###^*P* < 0.001 compared with P15; ^$^*P* < 0.05, ^$$^*P* < 0.01 compared with P20. Statistics are analyzed by one-way ANOVA and followed by Fisher's LSD test.

The developmental profile of EEG power spectra, EEG, EMG and sleep-wake states during dark (21:00–23:00 h) and light period (9:00–11:00 h) in P11-P75 rats were shown in Figure 5A. On the 11th day, the amount of REM sleep was very great (daily average 58.5 ± 1.4%) and its episode duration was short, and NREM occurrence was rare (daily average 5.1 ± 0.4%). The main transition between REM sleep and wakefulness was very quick so that the EEG power spectra displayed low density and scattering across the frequency of 0–30 Hz. P11-P15 pups still spent more time in REM than NREM sleep, though NREM sleep progressively increased and REM sleep decreased. The episode mean duration in each state progressively prolonged during this period. And interestingly, the immediate transition from wake to REM sleep occurred frequently. The amount of wakefulness and sleep between dark and light phase during the age from P11 to P19 was no obvious difference. However, the pups at P20 began to sleep more during light period than dark period. The diurnal rhythm of sleep-wake cycle at P30 was quite similar to that at P75. The EEG delta (0.5–4 Hz) power in NREM spectrum was markedly increased with age (Figure 5B). The stable amount, architecture and EEG power spectra across sleep-wake cycle during light and dark period were found in P30-P75 rats.

**Figure 6:**
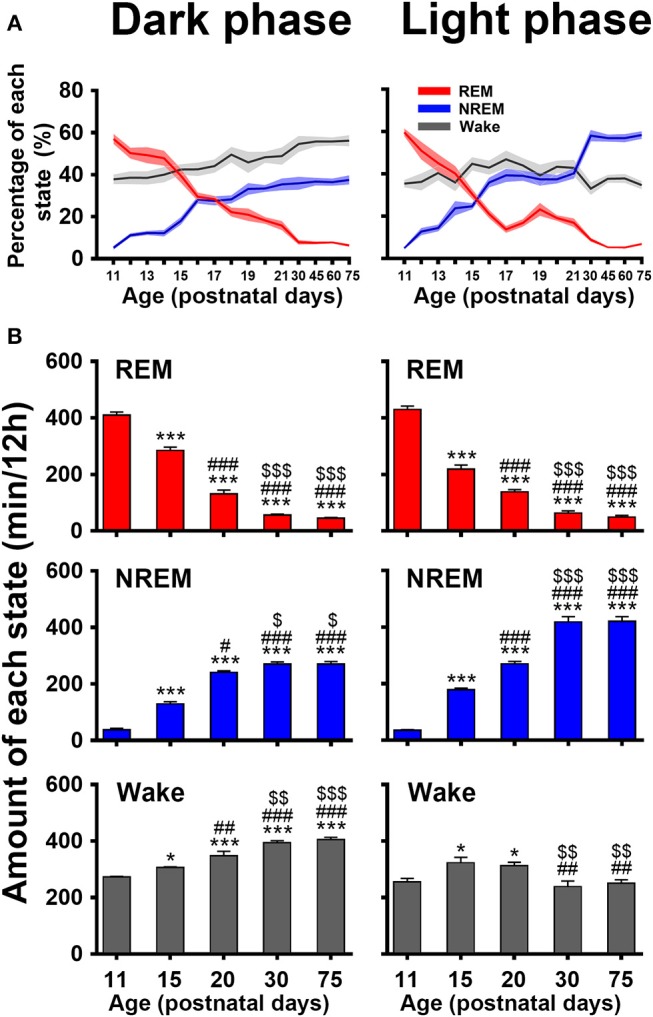
Developmental changes of percentage **(A)** and amount **(B)** of REM, and NREM sleep and wakefulness in dark and light phase from infant to adult rats. The amount analysis at the key postnatal days displayed that REM sleep dramatically decreased in both dark and light phases from P11 to P30, and NREM sleep and wakefulness meanwhile increased, but the amount of each state in respective dark and light phase from P30 to P75 was not obviously different. Notably, the increased NREM sleep in light phase was more than that in dark phase from P20 to P30, while wakefulness significantly increased in dark phase and decreased in light phase. Values are means ± SEM (*n* = 5–9 in each age), *P < 0.05, ***P < 0.001 compared with P11; ^#^*P* < 0.05, ^##^*P* < 0.01, ^###^*P* < 0.001 compared with P15; ^$^*P* < 0.05, ^$$^*P* < 0.01, ^$$$^*P* < 0.001 compared with P20. Statistics are analyzed by one-way ANOVA and followed by Fisher's LSD test.

The evolutional process of wake, NREM and REM sleep in dark and light phase from P11 to P75 was illustrated in Figure 6. The proportion of REM and NREM sleep had a dramatic decrease and increase, respectively (Figure 6A). The REM sleep was reduced respectively, from 57.0 ± 2.4 to 6.3 ± 0.6% in dark phase and from 59.7 ± 1.7 to 6.9 ± 0.5% in light phase. The NREM sleep in dark and light phase increased respectively, from 5.2 ± 0.8% to 37.5 ± 2.1% and from 4.9 ± 0.5 to 58.4 ± 1.7%. Wakefulness in dark phase from P11 to P75 increased from 37.8 ± 2.2 to 56.2 ± 2.6%. Attractively, the increased NREM sleep from P20 to P30 in light phase was more than that in the dark. Furthermore, an amount analysis of each state in dark and light phase at the considered key postnatal days (P11, P15, P20, P30, and P75; Figure 6B) during developmental process demonstrated that REM sleep from P11 to P30 dramatically decreased in both dark (410.2 ± 10.7 min vs. 56.2 ± 3.4 min, *P* < 0.001) and light (429.5 ± 12.4 min vs. 63.8 ± 7.1 min, *P* < 0.001) phases, while NREM sleep simultaneously increased in dark (37.6 ± 5.5 min vs. 270.5 ± 7.6 min, *P* < 0.001) and light phase (35.5 ± 1.9 min vs. 418.2 ± 18.7 min, *P* < 0.001). Notably, the increased NREM sleep from P20 to P30 in light phase was more than in dark phase (418.2 ± 18.7 min vs. 270.5 ± 7.6 min, *P* = 0.001). In the meantime, wakefulness significantly increased in dark phase (347.8 ± 15.8 min vs. 393.3 ± 7.7 min, *P* < 0.01) and decreased in light phase (312.4 ± 12.3 min vs. 238.0 ± 20.4 min, *P* < 0.01). However, the amount of each state in dark and light phase from P30 to P75 was not significantly different (*P* > 0.05).

**Figure 7:**
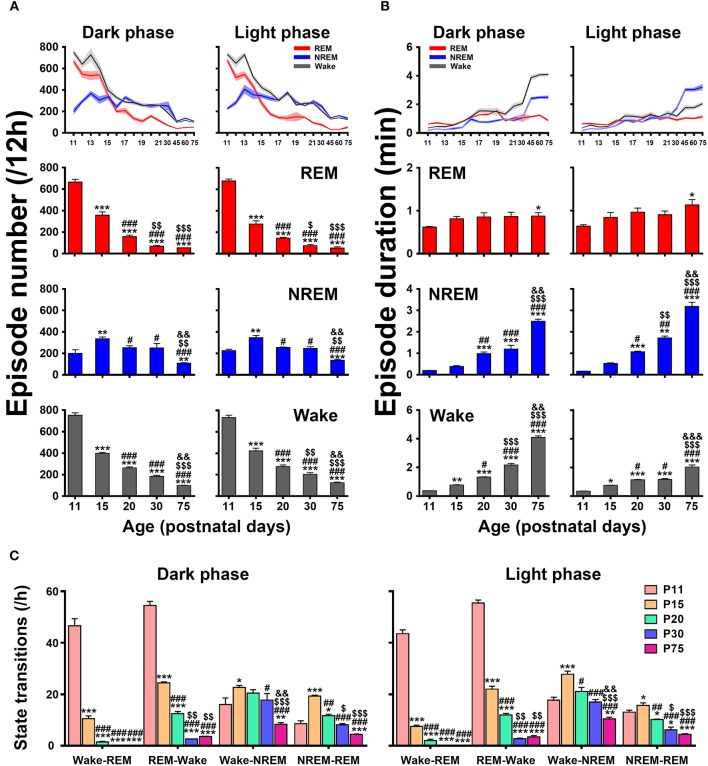
Developmental changes of episode number **(A)**, mean duration **(B)**, and state transitions **(C)** of REM, and NREM sleep and wakefulness in dark and light phase from infant to adult rats. The architectural analysis of three vigilance states at the key postnatal days showed that the episode number gradually declined in both dark and light phases with age and the mean duration of episode meanwhile increased, though NREM episode number temporarily increased at P15. The number of state transitions progressively decreased. Note that the transitions between wake and REM sleep sharply decreased from P11 to P20, and the transitions from wake to REM sleep were eventually absent in >P30 rats. Values are means ± SEM (*n* = 5–9 in each age), *P < 0.05, **P < 0.01, ***P < 0.001 compared with P11; ^#^*P* < 0.05, ^##^*P* < 0.01, ^###^*P* < 0.001 compared with P15; ^$^*P* < 0.05, ^$$^*P* < 0.01, ^$$$^*P* < 0.001 compared with P20; ^&&^*P* < 0.01, ^&&&^*P* < 0.001 compared with P30. Statistics are analyzed by one-way ANOVA and followed by Fisher's LSD test.

An architectural profile of sleep-wake development showed that the episode number of each state progressively decreased in both dark and light phases with age, while the mean episode duration significantly increased (upper panels in Figures 7A,B). A further statistical analysis at the key postnatal days (lower panels in Figures 7A,B) demonstrated that REM and wake episode number from P11 to P75 in dark phase (REM, 665.3 ± 26.6 vs. 52.6 ± 1.7 and wake, 752.0 ± 23.4 vs. 99.2 ± 2.1) and light phase (REM, 677.0 ± 18.8 vs. 52.1 ± 12.3 and wake, 733.0 ± 22.6 vs. 124.8 ± 5.8) were significantly reduced (*P* < 0.001), while their mean duration remarkably increased (REM, 0.62 ± 0.02 min vs. 0.88 ± 0.08 min, *P* < 0.05 and wake, 0.36 ± 0.01 min vs. 4.09 ± 0.11 min, *P* < 0.001 in dark phase; REM, 0.64 ± 0.04 min vs. 1.13 ± 0.13 min, *P* < 0.05 and wake, 0.35 ± 0.01 min vs. 2.04 ± 0.14 min, *P* < 0.001 in light phase). Notably, NREM episode number from P11 to P15 increased respectively, in dark (200.0 ± 34.6 vs. 336.0 ± 16.6, *P* < 0.01) and light phase (225.9 ± 12.3 vs. 345.6 ± 21.1, *P* < 0.01) and then decreased from P20 to P75 (251.8 ± 19.4 vs. 109.0 ± 4.2, *P* < 0.001 in dark phase and 254.1 ± 5.5 vs. 135.9 ± 9.4, *P* < 0.001 in light phase). Drastically, its episode duration from P20 to P75 was increased in both dark (0.98 ± 0.08 min vs. 2.50 ± 0.09 min, *P* < 0.001) and light phase (1.06 ± 0.04 min vs. 3.19 ± 0.19 min, *P* < 0.001). The analysis of state transitions (Figure 7C) demonstrated that the number of transitions of wake-REM (46.6 ± 2.8 in dark phase and 43.4 ± 1.5 in light phase) and REM-wake (54.4 ± 1.6 in dark phase and 55.3 ± 1.1 in light phase) were great at P11, and then sharply reduced and declined to a low level at P20 (wake-REM, 1.5 ± 0.2 in dark phase, *P* < 0.001 and 2.0 ± 0.6 in light phase, *P* < 0.001; REM-wake, 12.5 ± 0.8 in dark phase, *P* < 0.001 and 11.9 ± 0.6 in light phase, *P* < 0.001). And eventually the transitions of wake-REM was absent in >P30 rats. The transitions of wake-NREM and NREM-REM were increased in both dark and light phases at P15 compared to P11 and then gradually decreased.

## Discussion

In our study, the new approaches including two types of EEG electrodes, a temperature-controlled incubator and a milk-feeding system in freely moving infant rats were to successfully developed to perform continuous and distinguished EEG and EMG recordings, And it also enable us to distinctly unravel the ontogenetic features and architectures and cortical EEG power spectra across sleep-wake development. Our study demonstrated in P11 to P75 rats that they first (at P11) spent their most time in REM sleep in dark (57.0 ± 2.4%) and light phase (59.7 ± 1.7%) and less time in NREM sleep (5.2 ± 0.8 and 4.9 ± 0.5% respectively, in dark and light phase). And then REM sleep was sharply decreased with age and NREM sleep simultaneously increased. The dramatic evolution of reduction in REM sleep and of increase in NREM sleep mainly occurred between P11 and P30. Eventually, at P75 REM sleep reduced to 6.3 ± 0.6% in dark phase and 6.9 ± 0.5% in light phase, while NREM sleep increased up 37.5 ± 2.1% in dark phase and 58.4 ± 1.7% in light phase, respectively. EEG power spectra in 0.5–4 Hz increased with age accompanied with prolonged duration of cortical slow wave activity. Amount of sleep between light and dark phase were not obviously different until P20. The circadian rhythm of sleep-wake cycle in infants began to be comparable to adult rats from P30. The episode number of each state was gradually decreased with age in both dark and light phases, while the mean duration was significantly increased.

The feeding problem in the pup prior to weaning limits early sleep recording to only one or several hours a day. In the previous studies, formula milk was administered orally by syringe before sleep recording (2, 3, 17, 18, 20). The milk-feeding system used in our study was set up with a computer-assisted infusion pump and a conterminous PE tube connected to feeding catheter inserted into one side of oral cavity, and delivered milk once an hour (Figure 2). The programmable infusion pump could mimic the maternally periodic feeding throughout 24 h. And it allowed the pups to suckle the proper volume of milk at each corresponding age (Table 1) and eliminate the defect of manually unmanageable feeding at night (34). The temperature problem in maternally separated pups is another limitation for continuous PSG recording. The young pups are of poor adaptability to ambient temperature changes because their thermoregulatory system was immaturity (35, 36). When the ambient temperature decreases, the pups will experience a significant increase in wakefulness and decrease in sleep (22, 37). A temperature-controlled incubator was used in our study to keep the proper warmth of P9-P20 pups during recovery and PSG recording through setting temperature controller in line with the requirement of pup's age (Table 1) (20, 24, 27). Additionally, a potential probability of the changes of sleep-wake pattern caused by maternal separation before weaning (21) was also considered in our study. To induce micturition and defecation and reduce the stress induced by maternal deprivation (2, 23, 24), the pre-weaned pups were ministered to periodically groom and stimulate on the anogenital region. All in all, these approaches mentioned above employed in the present study largely ensured the growth, movement, eye-open time, gross appearance and weight gain (Figure 3) in maternally separated pups similar to those in control pups kept with their dam that sustained a continuously PSG recordings.

More importantly, the present study aims at an effective acquisition of cortical EEG and muscular EMG signals of the rat from neonatal to adult. This would contribute to the analysis of states and architectures during sleep-wake development. Gold-plated pins and screws served as EEG electrodes respectively, for P9-P16 and P17-P75 rats (Figure 1) accurately recorded a distinguishable EEG pattern of each state (Figure 4). The EMG activities of nuchal muscles in sleep-wake states at each age were stably recorded through sliver EMG electrodes (Figure 4). The innovation of 4-pins EEG electrodes assembled within a 6-pin pedestal used in P9-P16 pups was considered to be light and perfectly fit the small, soft and fragile skull surface. In addition, the infrared video camera was used during recovery after surgery and PSG recording through which to monitor the behavioral alterations in state development. The occurrences of myoclonic twitches, the discrete and spontaneous limb movements exclusively during REM sleep in early development that contribute to distinguish sleep-wake states in young pups (38, 39) were also observed in this study. Thus, the evolutional characteristics of both EEG and EMG activities and cortical EEG power spectra, and behavioral states were identified perfectly in ontogeny of sleep-wake states in dark and light phase (Figure 5).

Using the novel methods, the development information of sleep-wake states in P11-P75 rats was acquired in the present study. The developmental profiles showed that the amount of REM was 410.2 ± 10.7 min in dark phase and 429.5 ± 12.4 min in light phase at P11. For rats at P30, the REM sleep was dramatically declined to 56.2 ± 3.4 min (*P* < 0.001) in dark phase and 63.8 ± 7.1 min (*P* < 0.001) in light phase, respectively. Conversely, NREM sleep between P11 and P30 increased from 37.6 ± 5.5 min to 270.5 ± 7.6 min (*P* < 0.001) in dark phase and from 35.5 ± 1.9 min to 418.2 ± 18.7 min (*P* < 0.001) in light phase. Wakefulness between P11 and P30 significantly increased in both dark and light phases, except it decreased from P20 to P30 in light phase. The amount of each state in respective dark and light phase from P30 to P75 was not obviously different (Figure 6). The evidences indicate that the dramatic changes of sleep-wake cycle mainly occur in the first month of postnatal rats. Additionally, the pups at P20 began to sleep more during light period than dark period. And the diurnal rhythm of sleep-wake cycle built up completely at P30. The high amount and intensity of REM sleep in early life of mammals has been observed in previous studies (1, 3, 27, 29) that is proposed to serve as an indicator for the degree of brain maturation and the promoter of further brain development (40–42). Higher levels of REM sleep are needed by altricial mammals such as neonatal rats, which are born with relatively shorter gestational time and thus require relatively more brain development to reach adulthood (43). The decrease of REM sleep with age indicates the maturation of inhibitory mechanism of REM sleep generator center (41, 42). NREM sleep is less in early life. Actually, it does not show up until the brain has developed a certain maturity (3, 44). The dramatic increase of NREM sleep with age is considered to parallel cortical development including synaptic connections, energy use and metabolic homeostasis (45), though its mechanism involved in the brain maturation remains to elucidate. The diurnal rhythm of sleep-wake cycle emerged at the third postnatal week, suggesting that rats are born with an immature circadian system, which achieves its completion during postnatal development. Despite the presence of endogenous rhythms of the suprachiasmatic nucleus in the mammalian fetus, newborn animals do not display circadian organization (27, 45).

The study also demonstrated the evolutional features of sleep-wake architectures. The episode number of three vigilance states was progressively decreased with age, while the mean episode duration of that was significantly increased (Figures 7A,B). Notably, the mean duration of NREM episode from P20 to P75 was increased in light phase more than in the dark. The number of state transitions was great in neonatal rats and then decreased with age (Figure 7C). The transitions between wake and REM sleep predominated at P11, and then sharply declined and eventually absented in > P30 rats. The characteristics of state transitions in infant rats are similar to that have been observed in early life in humans (1). The transitions between NREM and REM sleep, and wake and NREM sleep were greater at P15 and then decreased with age, suggesting that NREM sleep tardily forms than REM sleep. These results indicate that rats, similar to humans, have a dramatic change in sleep architecture in the first month (27, 45). The consolidation of sleep and waking episodes throughout development is gradual evolution through increasing the episode duration and decreasing the episode number or quick transitions among three vigilance states (27).

In conclusion, using special EEG and EMG electrodes in maternally separated infant rats, and supported by the milk-feeding system and temperature-controlled incubator, we successfully record the continuous and distinguished EEG and EMG signals. And this allows us to analyze the ontogenetic profiles, architecture and EEG power spectra of sleep-wake states. REM sleep predominates in early developmental sleep and subsequently reduces with age. NREM sleep is of very little amount in neonatal rats, and then augments accompanied with EEG delta activity with age. And finally it predominates in lately developmental sleep. In addition, episode number and duration of each state decreases and increases respectively, with age. The above dramatic changes occur mainly in the first month after birth, suggesting that REM and NREM sleep are required in developmental node from immature to mature brain, and play different role in different stages. Thus, the innovative approaches settle a fundamental method for unraveling the developmental relationship between sleep and brain in future.

## Data Availability

All datasets generated for this study are included in the manuscript and/or the supplementary files.

## Ethics Statement

This study was carried out in accordance with the National Institutes of Health Guide for the Care and Use of Laboratory Animals (2011 revision). The experimental protocol was approved by the Ethics Committee of Lanzhou University (permit number: SCXK Gan 2018-0002, Lanzhou, PR China).

## Author Contributions

Y-PH, G-FC, MH, and Y-FS designed the study. G-FC, MH, H-LC, Y-NC, and J-XG conducted the experiments. C-YC and F-QD collected and analyzed the data. J-FX conducted statistical analysis. Y-PH, G-FC, and Y-FS wrote the paper. All authors approved the final version and evaluated the accuracy and integrity of the work.

### Conflict of Interest Statement

The authors declare that the research was conducted in the absence of any commercial or financial relationships that could be construed as a potential conflict of interest.
